# Alterations of brain white matter network topological properties in overt hypothyroidism

**DOI:** 10.1530/EC-25-0039

**Published:** 2025-05-16

**Authors:** Jinghe Tian, Quan Zou, Jiancang Cao, Liting Wang, Jing Tian, Chen Yang, Wenxiu Ma, Gang Huang, Jian Tan, Wenwen Zhang, Lianping Zhao

**Affiliations:** ^1^ The First Clinical Medical College, Gansu University of Chinese Medicine (Gansu Provincial Hospital), Lanzhou, Gansu, China; ^2^ Department of Radiology, Gansu Provincial Hospital, Lanzhou, Gansu, China

**Keywords:** overt hypothyroidism, white matter network, cognition, emotion

## Abstract

**Purpose:**

This study examined the topological properties of brain white matter networks in overt hypothyroidism (OH) patients and their links to cognitive and emotional dysfunction.

**Materials and methods:**

Fifty OH patients and 92 healthy controls underwent brain magnetic resonance imaging, clinical assessments and neuropsychological evaluations. Graph-theoretical network analysis based on diffusion tensor imaging was used to calculate global and local topological properties. Between-group differences were analyzed, and partial correlation and mediation analyses were conducted to explore relationships among topological metrics, clinical variables and neuropsychological scores.

**Results:**

The OH group showed significantly higher depressive and anxious scores, and lower cognitive scores. In the global topological analysis, the OH group showed decreased global efficiency, which was negatively correlated with the Hamilton Rating Scale for Depression-24 scores. Local topological abnormalities were predominantly observed in the nodal efficiency (NE), degree centrality and nodal local efficiency of several regions within the limbic system and default mode networks. Notably, NE in the left amygdala and left paracentral lobule was negatively correlated with the Hamilton Rating Scale for Depression-24 scores, and decreased NE in the right median cingulate and paracingulate gyri was positively correlated with executive function/visuospatial ability scores and the clock drawing test score.

**Conclusion:**

OH patients show depression, anxiety and cognitive impairments linked to global efficiency and regional abnormalities in the limbic system and default mode network. These findings provide insights into the neuropathophysiological mechanisms underlying emotional and cognitive impairments.

## Introduction

Hypothyroidism, a prevalent endocrine disorder worldwide (1), is defined by insufficient production of thyroid hormones or reduced hormonal action in target tissues, leading to a systemic hypometabolic state. It is characterized by increased thyroid-stimulating hormone (TSH) level and decreased free thyroxine (FT4) level (2). Previous studies have indicated that hypothyroidism is frequently associated with cognitive impairments and emotional abnormalities (3, 4). Without timely and effective intervention, patients may face an increased risk of developing dementia (5) and depression (6, 7), significantly reducing their quality of life. However, the underlying neuropathophysiological mechanisms contributing to these cognitive and emotional disturbances remain inadequately understood.

Advancements in neuroimaging technologies have provided novel insights into the cerebral effects of hypothyroidism. Functional and structural magnetic resonance imaging (MRI) have been utilized to investigate brain functional and structural changes in patients with hypothyroidism. Studies have reported decreased connectivity between the default mode network (DMN) and the frontoparietal attention network (8), decreased hippocampal volume (9), and fractional anisotropy (FA) that was significantly lower in the cerebellum and right temporal lobe in hypothyroid patients (10). In recent years, our research group has found increased functional connectivity between the cerebellum and specific regions in the frontal and parietal lobes (11), as well as decreased hippocampal volume and changes in hippocampal and whole-brain functional connectivity (12) in hypothyroid patients. Furthermore, hypothyroid patients’ microstructural impairments to multiple white matter (WM) fiber tracts – including the corticospinal tract and inferior longitudinal fasciculus – have been associated with cognitive decline and depressive symptoms (13, 14). Given that brain WM consists of myelinated neuronal axons crucial for maintaining normal brain function, alterations in WM integrity or changes in the topological properties of brain networks can significantly influence the transmission and processing of neural information. Graph-theoretical analyses based on diffusion tensor imaging (DTI) data have emerged as a powerful approach for investigating the topological properties of brain WM networks, revealing changes that are closely related to information transmission and functional integration. This method offers a novel means of quantifying the overall structural connectivity patterns of the brain (15, 16) and has been widely applied in studying various conditions associated with cognitive deficits and psychological disorders, such as Alzheimer’s disease and depression (17, 18). However, research on the topological properties of brain WM networks in overt hypothyroidism (OH) patients is limited, leaving it unclear how these properties are altered in this population.

Therefore, the present study aims to collect clinical data, structural MRI data and neuropsychological scale scores from patients with OH and healthy controls (HCs) to comparatively analyze changes in the topological properties of the brain WM network. In addition, we seek to examine the relationships between these changes and cognitive dysfunction as well as emotional abnormalities. We hypothesize that OH patients exhibit altered global and local topological properties in the brain WM network, particularly in regions involved in higher-order cognitive and emotional processing. These alterations may correlate with neuropsychological scale scores and levels of thyroid-related hormones and blood lipids.

## Materials and methods

### Participants

The study prospectively recruited 51 OH patients from the endocrinology department of Gansu Provincial Hospital between October 2019 and December 2023. In addition, 94 HCs matched for age and years of education were recruited through advertisements. The study adhered to the Declaration of Helsinki and was approved by the Gansu Provincial Hospital Medical Ethics Committee (batch number: 2019-196 and 2023-500). Inclusion criteria were as follows: i) age 18–60 years; ii) at least 6 years of education; iii) Chinese Han ethnicity; iv) right-handed; v) the OH group consisted of patients who had not received treatment at the time of the initial diagnosis and met the diagnostic criteria of the 2017 edition of the Adult Guidance for Diagnosis and Treatment of Hypothyroidism. The specific biochemical criteria were an elevation of serum TSH levels above the reference range (0.35–4.94 mIU/L) and reduced serum FT4 levels below the reference value (9.01–19.05 pmol/L) (6, 19); controls with TSH, FT4, free triiodothyronine (FT3), triiodothyronine (T3) and thyroxine (T4) levels within the normal range were recruited from the surrounding communities in the HCs group, with the Hamilton Rating Scale for Depression-24 (HAMD-24) scores <8, Hamilton Anxiety Rating Scale (HAMA) scores <7 and Montreal Cognitive Assessment (MoCA) scores ≥26. Exclusion criteria included: i) history of psychiatric disorders or related family history; ii) history of craniocerebral trauma or surgery; iii) severe hearing or visual impairment; iv) alcohol dependence or drug abuse; v) pregnant, lactating, or those taking oral contraceptives; vi) use of psychotropic medication for at least 2 months or received treatment with transcranial magnetic stimulation in the last 3 months; vii) other endocrine diseases (such as congenital hypothyroidism, hyperthyroidism, Cushing’s disease or diabetes); viii) any contraindications to MRI examination. Those who met the inclusion criteria were scheduled for an MRI scan on the same day to ensure that patients could start their medication regimen promptly after the scan.

### Demographic data, clinical variables and neuropsychological assessments

Sex, age and years of education were documented for the participants. Venous blood samples were collected after overnight fasting to assess thyroid function and blood lipid levels. Thyroid function tests included measurements of TSH, T3, T4, FT3, FT4, thyroglobulin antibodies (TgAb) and thyroid peroxidase antibodies (TPOAb). Blood lipid tests included serum triglyceride, cholesterol (CHOL), low-density lipoprotein cholesterol (LDL) and high-density lipoprotein cholesterol. In addition, all subjects were evaluated for depression and anxiety symptoms using the HAMD-24 scale and the HAMA scale, and cognitive functions were evaluated using the MoCA scale.

### MRI data acquisition

Whole-brain MRI scans were acquired using a 3.0 T scanner (MAGNETOM Skyra, Siemens Healthcare, Germany) equipped with a 32-channel phased-array head coil. Participants were asked to keep their heads still throughout the scanning period. Conventional MRI sequences included axial T1-weighted imaging, T2-weighted imaging and T2 fluid-attenuated inversion recovery images. Scanning and diagnosis were performed by two experienced radiologists to exclude any anatomical abnormalities and organic lesions. High-resolution three-dimensional T1-weighted imaging (3D-T1WI) and DTI were then acquired. The 3D-T1WI sequence parameters were: repetition time = 2,530 ms; echo time = 2.35 ms; inversion time = 1,100 ms; flip angle = 7°; matrix size = 256 × 256; field of view = 256 × 256 mm^2^; slice thickness = 1.33 mm; slices = 192; acquisition time = 5 min and 23 s. DTI data were acquired using a spin-echo echo-planar imaging sequence with: repetition time = 11,300 ms; echo time = 85 ms; matrix size = 224 × 224; field of view = 224 × 224 mm^2^; slice thickness = 3.5 mm; slices = 64; voxel size = 2 × 2 × 2.5 mm^3^; acquisition time = 12 min and 8 s; diffusion weighting with both 64 directions (*b* = 1,000 s/mm^2^) and one non-diffusion-weighted volume (*b* = 0 s/mm^2^).

### Processing of cranial MRI data

#### Preprocessing of MRI data

First, to perform origin correction on the 3D-T1WI image, the SPM12 tool (http://www.fil.ion.ucl.ac.uk/spm/), which operates within the MATLAB environment, was used. Next, the Pipeline for Analyzing Brain Diffusion Images Toolkit (https://www.nitrc.org/projects/panda), developed by the State Key Laboratory of Cognitive Neuroscience and Learning at Beijing Normal University, was utilized for standardized preprocessing of DTI data. This toolkit is a MATLAB-based toolbox that leverages the FMRIB Software Library (FSL, http://www.fmrib.ox.ac.uk/fsl/). The preprocessing workflow included: i) conversion to NIFTI format: DICOM images were converted using the dcm2nii tool in FSL; ii) eddy current and motion correction: image distortions and head movements were corrected; iii) brain extraction: a brain mask was obtained from non-diffusion-weighted images (*b* = 0 s/mm^2^) using the brain extraction tool; iv) FA map generation: FA maps were extracted by fitting the diffusion tensor for each voxel using a linear least-squares algorithm with the DTIFIT tool for subsequent construction of the WM network.

#### Construction of brain white matter network

In this study, we used the FA map to construct the WM network. First, network nodes were constructed from 90 nodes (45 regions for each hemisphere) segmented by the automated anatomical labeling (AAL) (20) atlas, which has been widely used in structural brain network analyses (21). Individual 3D-T1WI images were co-registered to the *b* = 0 images and normalized to the Montreal Neurological Institute (MNI) 152 space. The AAL atlas was then inversely transformed from MNI space to each subject’s DTI space, resulting in individualized AAL atlases. Whole-brain WM fiber tracking was performed using a deterministic continuous tracking algorithm. Fiber tracking was terminated when the deflection angle exceeded 45° or when the FA value of a voxel dropped below 0.2 (22). All fiber tracts connecting each pair of brain regions were extracted. The average FA value was used as the connected edge of the network, and a 90 × 90 matrix was generated using nodes and connected edges to represent the FA-weighted WM network of each subject.

#### Calculation of network topological metrics

Network metrics were calculated using the GRETNA toolbox (http://www.nitrc.org/projects/gretna/) developed by the State Key Laboratory of Cognitive Neuroscience and Learning at Beijing Normal University. The BrainNet Viewer (http://www.nitrc.org/projects/bnv/) was employed for visualization of different brain regions. Calculated metrics included global topological properties such as small-worldness, global efficiency (Eg), and assortativity, as well as nodal topological properties including betweenness centrality, degree centrality (DC), nodal clustering coefficient, nodal efficiency (NE), nodal local efficiency (NLe) and nodal shortest path. Based on previous studies, the sparsity threshold range was set from 0.10 to 0.30 with an interval of 0.01 (23), and 1,000 random network iterations were performed.

### Statistical analysis

Statistical analyses of demographic data, clinical variables and neuropsychological scale scores were conducted using SPSS software (version 26.0, IBM Corp., USA). Continuous variables were tested for normality. Data conforming to a normal distribution – including age, years of education, CHOL, high-density lipoprotein cholesterol, MoCA total score and MoCA subitems (executive functions/visuospatial abilities, clock drawing test, naming, attention, digit span forward and backward, serial sevens test, language, sentence repetition and abstraction) – are presented as mean ± standard deviation and were analyzed using two-sample *t*-tests. Data not conforming to a normal distribution – including TSH, T3, T4, FT3, FT4, TgAb, TPOAb, triglyceride, HAMD-24, HAMA and MoCA subitems such as the modified trail making test, copy of the cube, vigilance (letter tapping test), letter fluency and delayed recall – were presented as median (interquartile range) and were analyzed using the Mann–Whitney U test. Categorical data (sex) were assessed using the *χ*^2^ test. *P* < 0.05 was considered statistically significant.

The image data underwent processing to derive the mean FA value of the connected fibers between each pair of nodes. Fisher *r*-*to*-*z* transformation was applied to improve data normality for subsequent analyses. Using the GRETNA toolbox, two-sample *t*-tests were conducted to compare global topological properties (small-worldness, Eg and assortativity) and the nodal topological properties (betweenness centrality, DC, nodal clustering coefficient, NE, NLe, nodal shortest path) between groups, with sex, age and years of education included as covariates. Multiple comparisons were corrected using the false discovery rate (FDR). Results were visualized using the R programming language (https://www.r-project.org/).

Significantly different network topological metrics were extracted. Partial correlation analyses were performed between these metrics and neuropsychological scale scores, thyroid hormone levels and blood lipid levels in OH patients, controlling for sex, age and years of education. Mediation analyses were further performed to explore whether clinical variables indirectly contribute to cognitive and emotional abnormalities by affecting network topological metrics.

## Results

### Demographic data, clinical variables and neuropsychological assessments

Due to incomplete imaging data, one OH patient and two HCs were excluded. The final analysis included 142 participants (OH: *n* = 50, HCs: *n* = 92). The OH group had significantly higher serum levels of TSH, LDL, CHOL, TgAb and TPOAb than the HCs group, but significantly lower levels of T3, FT3, T4 and FT4. Detailed demographic data and clinical variables are shown in Table 1. In neuropsychological assessments, the OH group demonstrated lower scores on the MoCA total score and subitems including executive functions/visuospatial abilities, clock drawing test, attention, serial sevens test, vigilance (letter tapping test) and delayed recall scores. In addition, OH patients had higher scores on the HAMD-24 and HAMA. The detailed neuropsychological assessments are shown in Table 2. The differences in demographic and clinical variables after sex matching were fully aligned with those reported in this study. Apart from the lack of intergroup differences in neuropsychological scores for executive functions/visuospatial abilities, the clock drawing test, and vigilance (letter tapping test), all other intergroup differences in MoCA total score, attention, serial sevens test, delayed recall, and both HAMD-24 and HAMA scores were still significant (Supplementary Tables S2 and S3, see section on supplementary materials given at the end of this article).

**Table 1:** The demographic data and clinical variables of the two groups (HCs and OH).

	Reference range	HCs (*n* = 92)	OH (*n* = 50)	*χ^2^/t/z* value	*P* value
Sex (M/F)		34/58	10/40	4.356*	**0.037**
Age (years)		39.63 ± 10.72	40.80 ± 10.31	−2.242^†^	0.809
Education (years)		13.88 ± 3.43	13.08 ± 3.75	1.284^†^	0.201
TSH (mIU/L)	0.35–4.94 mIU/L	1.97 (1.18)	61.27 (64.52)	−9.827^‡^	**<0.001**
T3 (nmol/L)	0.98–2.33 nmol/L	1.55 (0.42)	1.07 (0.74)	−6.839^‡^	**<0.001**
T4 (nmol/L)	62.68–150.84 nmol/L	98.60 (20.99)	34.05 (40.70)	−9.766^‡^	**<0.001**
FT3 (pmol/L)	2.43–6.01 pmol/L	4.42 (0.64)	2.77 (1.38)	−7.802^‡^	**<0.001**
FT4 (pmol/L)	9.01–19.05 pmol/L	13.07 (2.06)	5.77 (2.45)	−9.239^‡^	**<0.001**
CHOL (mmol/L)	<5.2 mmol/L	4.33 ± 0.79	5.30 ± 1.19	−5.188^†^	**<0.001**
TG (mmol/L)	<1.7 mmol/L	1.12 (0.85)	1.22 (0.75)	−1.877^‡^	0.06
HDL (mmol/L)	>1.0 mmol/L	1.21 ± 0.27	1.32 ± 0.28	−2.389^†^	0.018
LDL (mmol/L)	<3.4 mmol/L	2.54 ± 0.66	3.24 ± 0.82	−5.550^†^	**<0.001**
TgAb (IU/mL)	<4.11 IU/mL	1.21 (2.43)	226.17 (970.1)	−8.463^‡^	**<0.001**
TPOAb (IU/mL)	<5.61 IU/mL	0.68 (1.26)	771.99 (820.46)	−8.724^‡^	**<0.001**
Duration (days)			30 (178)		

* Represents the *χ*^2^ value.

^†^ Represents the *t* value (two-sample *t*-test).

^‡^ Represents the *z* value (Mann–Whitney U test).

Abbreviations: HCs, healthy controls; OH, overt hypothyroidism; TSH, thyroid-stimulating hormone; T4, thyroxine; T3, triiodothyronine; FT3, free triiodothyronine; FT4, free thyroxine; CHOL, cholesterol; TG, triglyceride; HDL, high-density lipoprotein; LDL, low-density lipoprotein; TgAb, anti-thyroglobulin antibodies; TPOAb, thyroid peroxidase antibodies. Bold indicates statistical significance.

**Table 2:** Neuropsychological assessments of the two groups (HCs and OH).

	HCs (*n* = 92)	OH (*n* = 50)	*t/z* value	*P* value
MoCA	27.06 ± 2.66	25.44 ± 3.29	3.181*	**0.002**
Subdomains of MoCA				
Visuospatial/executive	4.26 ± 1.03	3.90 ± 1.05	2.015*	**0.046**
Modified trail making test	1.00 (0.00)	1.00 (1.00)	−1.900^†^	0.057
Copy of the cube	1.00 (0.00)	1.00 (0.00)	−0.211^†^	0.883
Clock drawing test	2.66 ± 0.63	2.42 ± 0.67	2.094*	**0.038**
Naming	2.94 ± 0.23	2.90 ± 0.30	0.949*	0.346
Attention	5.72 ± 0.56	5.26 ± 1.12	2.706*	**0.009**
Digit span forward and backward	1.86 ± 0.34	1.83 ± 0.37	0.470*	0.639
Vigilance (letter tapping test)	1.00 (0.00)	1.00 (0.00)	−1.992^†^	**0.046**
Serial sevens test	2.95 ± 0.23	2.66 ± 0.77	2.556*	**0.013**
Language	2.57 ± 0.66	2.60 ± 0.66	−0.255*	0.799
Sentence repetition	1.71 ± 0.55	1.75 ± 0.55	−0.386*	0.700
Letter fluency	1.00 (0.00)	1.00 (0.00)	−0.159^†^	0.873
Abstraction	1.90 ± 0.42	1.77 ± 0.42	1.712*	0.089
Delayed recall	4.00 (2.50)	3.00 (2.00)	−3.067^†^	**0.002**
Orientation	5.95 ± 0.20	5.96 ± 0.20	−0.129*	0.897
HAMD-24	2.00 (4.75)	6.00 (6.25)	−4.327^†^	**<0.001**
HAMA	1.00 (2.00)	4.00 (7.75)	−5.317^†^	**<0.001**

* Represents the *t* value (two-sample *t*-test).

^†^ Represents the *z* value (Mann–Whitney U test).

Abbreviations: HCs, healthy controls; OH, overt hypothyroidism; MoCA, Montreal Cognitive Assessment; HAMD-24, Hamilton Rating Scale for Depression-24; HAMA, Hamilton Anxiety Rating Scale. Bold indicates statistical significance.

### Global topological properties

**Figure 1:**
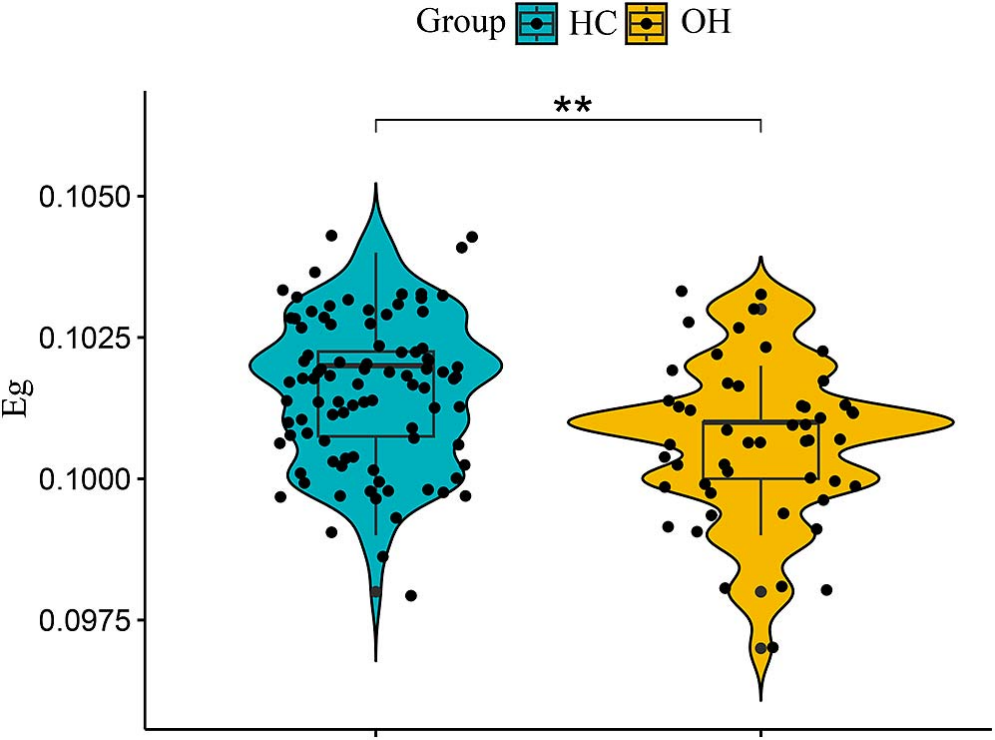
Intergroup differences of Eg between the two groups. *Represents *P* < 0.05; **represent *P* < 0.01; ***represent *P* < 0.001. Abbreviations: healthy controls (HCs); overt hypothyroidism (OH); global efficiency (Eg).

Both HCs and OH patients exhibited small-worldness in their WM network (sigma >1.2), with no significant differences in sigma value between groups (*P* > 0.05). However, Eg was significantly decreased in the OH group compared to HCs (*P* < 0.001). The differences in Eg between the two groups are illustrated in Fig. 1. Even after sex matching, the statistically significant differences in Eg between the two groups persisted (Supplementary Figure S1).

### Local topological properties

**Figure 2:**
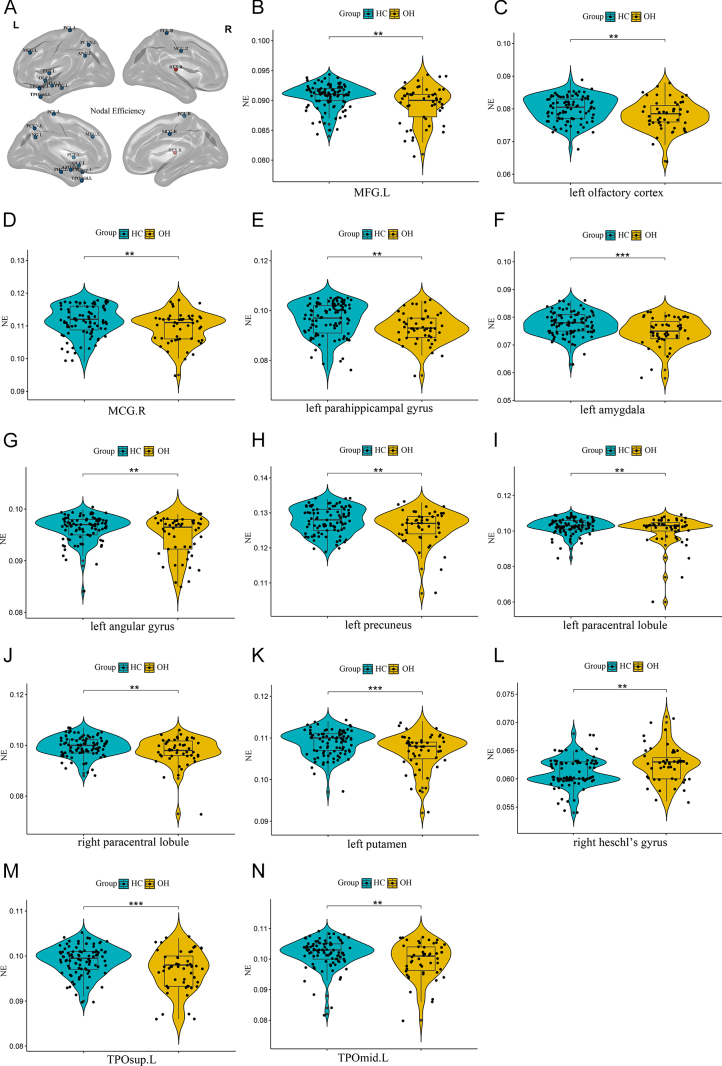
(A) Figure showing brain regions with between-group differences in NE values. Regions with decreased in NE are shown with blue nodes. Regions with increased NE are shown with red nodes (B, C, D, E, F, G, H, I, J, K, L, M, N). Intergroup differences in NE values of MFG.L, left olfactory cortex, MCG.R, left parahippocampal gyrus, left amygdala, left angular gyrus, left precuneus, left paracentral lobule, right paracentral lobule, left putamen, right Heschl’s gyrus, TPOsup.L and TPOmid.L between the two groups. *Represents *P* < 0.05; **represent *P* < 0.01; ***represent *P* < 0.001. Abbreviations: healthy controls (HCs); overt hypothyroidism (OH); left middle frontal gyrus (MFG.L); right median cingulate and paracingulate gyri (MCG.R); left temporal pole (superior temporal gyrus) (TPOsup.L); left temporal pole (middle temporal gyrus) (TPOmid.L); nodal efficiency (NE).

**Figure 3:**
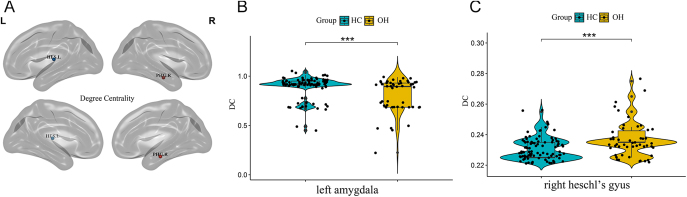
(A) Figure showing brain regions with between-group differences in DC values. Regions with decreased NE are shown with blue nodes. Regions with increased NE are shown with red nodes. (B and C) Intergroup differences in DC values of the left amygdala and right Heschl’s gyrus between the two groups. *Represents *P* < 0.05; **represent *P* < 0.01; ***represent *P* < 0.001. Abbreviations: healthy controls (HCs); overt hypothyroidism (OH); degree centrality (DC).

**Figure 4:**
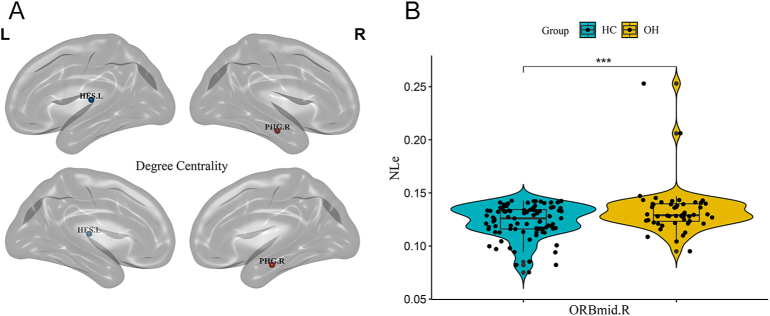
(A) Figure showing brain regions with between-group differences in NLe values. (B) Intergroup differences in NLe values of ORBmid.R between the two groups. *Represents *P* < 0.05; **represent *P* < 0.01; ***represent *P* < 0.001. Abbreviations: healthy controls (HCs); overt hypothyroidism (OH); right orbital part of middle frontal gyrus (ORBmid.R); nodal local efficiency (NLe).

Significant alterations were observed in the NE of several brain regions in the OH group compared to HCs. Specifically, NE was decreased in 12 brain regions: left middle frontal gyrus (*t* = 2.97, *P* = 0.004), left olfactory cortex (*t* = 2.78, *P* = 0.006), right median cingulate and paracingulate gyri (MCG.R) (*t* = 3.04, *P* = 0.003), left parahippocampal gyrus (*t* = 3.10, *P* = 0.003), left amygdala (*t* = 4.34, *P* < 0.001), left angular gyrus (*t* = 3.10, *P* = 0.002), left precuneus (*t* = 2.86, *P* = 0.005), left paracentral lobule (*t* = 2.89, *P* = 0.005), right paracentral lobule (*t* = 3.20, *P* = 0.002), left putamen (*t* = 3.66, *P* < 0.001), left temporal pole (superior temporal gyrus) (TPOsup.L) (*t* = 3.67, *P* < 0.001) and left temporal pole (middle temporal gyrus) (TPOmid.L) (*t* = 3.03, *P* = 0.003). Conversely, NE was significantly increased in the right Heschl’s gyrus (*t* = −2.87, *P* = 0.005) in the OH group. These alterations are depicted in Fig. 2. Regarding DC, OH patients showed decreased DC in the left amygdala (*t* = 4.16, *P* < 0.001) but increased DC in the right Heschl’s gyrus (*t* = −3.90, *P* < 0.001). These alterations are depicted in Fig. 3. NLe was increased in the right orbital part of the middle frontal gyrus (ORBmid.R) (*t* = −3.91, *P* < 0.001). This alteration is depicted in Fig. 4. All differences in local topological property metrics have been corrected using FDR. After sex matching, we found that the inter-group differences in the left olfactory cortex NE and the left precuneus NE disappeared, while differences in the left insula NE became apparent. The other differences in local topological indicators (FDR) remained consistent with those found in this study (Supplementary Figure S2).

### Partial correlation between topological property metrics and neuropsychological scale scores

**Figure 5:**
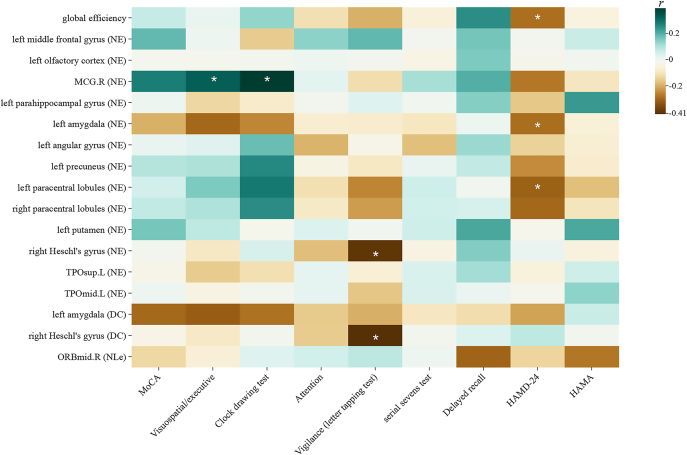
Partial correlation analysis between neuropsychological scale scores and differential topological property metrics. *Represents *P* < 0.05; **represent *P* < 0.01; ***represent *P* < 0.001. Abbreviations: right median cingulate and paracingulate gyri (MCG.R); left temporal pole (superior temporal gyrus) (TPOsup.L); left temporal pole (middle temporal gyrus) (TPOmid.L); right orbital part of middle frontal gyrus (ORBmid.R); nodal efficiency (NE); degree centrality (DC); nodal local efficiency (NLe); Montreal Cognitive Assessment (MoCA); Hamilton Rating Scale for Depression-24 (HAMD-24); Hamilton Anxiety Rating Scale (HAMA).

Partial correlation analyses were conducted between altered topological property metrics and neuropsychological scale scores within the OH group, controlling for sex, age and years of education. The HAMD-24 scores negatively correlated with Eg (*r* = −0.290, *P* = 0.048), NE of the left amygdala (*r* = −0.291, *P* = 0.049), and NE of the left paracentral lobule (*r* = −0.314, *P* = 0.048). There were positive correlations between the NE of the MCG.R and executive functions/visuospatial abilities scores (*r* = 0.327, *P* = 0.044), and clock drawing test score (*r* = 0.392, *P* = 0.021). In addition, the NE value of the right Heschl’s gyrus (*r* = −0.403, *P* = 0.026) and the DC value of the right Heschl’s gyrus (*r* = −0.416, *P* = 0.028) were negatively correlated with vigilance score. These results of the correlation analysis are shown in Fig. 5. All of the above results were FDR corrected.

### Partial correlation between topological property metrics and clinical variables

**Figure 6:**
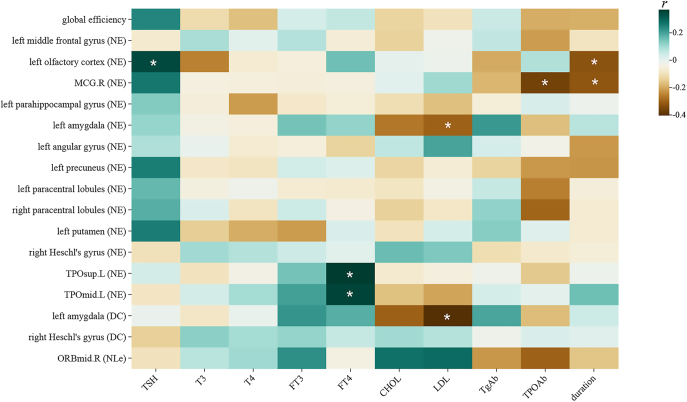
Partial correlation analysis between clinical variables and differential topological property metrics. *Represents *P* < 0.05; **represent *P* < 0.01; ***represent *P* < 0.001. Abbreviations: right median cingulate and paracingulate gyri (MCG.R); left temporal pole (superior temporal gyrus) (TPOsup.L); left temporal pole (middle temporal gyrus) (TPOmid.L); right orbital part of middle frontal gyrus (ORBmid.R); nodal efficiency (NE); degree centrality (DC); nodal local efficiency (NLe); thyroid-stimulating hormone (TSH); triiodothyronine (T3); thyroxine (T4); free triiodothyronine (FT3); free thyroxine (FT4); cholesterol (CHOL); low-density lipoprotein (LDL); thyroglobulin antibodies (TgAb); thyroid peroxidase antibodies (TPOAb).

Partial correlation analyses were conducted between altered topological property metrics and clinical variables within the OH group, controlling for sex, age and years of education. Positive correlations: the NE of the left olfactory cortex and TSH (*r* = 0.346, *P* = 0.036), the NE of the TPOsup.L and FT4 (*r* = 0.369, *P* = 0.033), and the NE of the TPOmid.L and FT4 (*r* = 0.355, *P* = 0.033). Conversely, the NE of the MCG.R and TPOAb (*r* = −0.369, *P* = 0.032). In addition, there were negative correlations between LDL and the NE of the left amygdala (*r* = −0.300, *P* = 0.048), and the DC of the left amygdala (*r* = −0.404, *P* = 0.021). We also observed negative correlations between the NE of the left olfactory cortex and duration (*r* = −0.328, *P* = 0.048), and between the NE of the MCG.R and duration (*r* = −0.322, *P* = 0.044). The correlation analyses are presented in Fig. 6. All of the above results were FDR corrected. Further, stepwise multiple linear regression analyses revealed that LDL levels exerted a significant influence on changes in NE and DC of the left amygdala. Additionally, FT4 levels significantly influenced changes in NE of the TPOsup.L and TPOmid.L (Supplementary Table S1).

### Mediation analysis

Mediation analysis demonstrated that abnormal changes in WM network topological properties did not mediate the relationship between clinical variables (e.g., hormonal and lipid levels) and emotional or cognitive abnormalities.

## Discussion

This study investigated alterations in the topological properties of the WM network in OH patients using graph-theoretical analysis of DTI data. Our main findings are threefold: i) Although the WM network of the OH group retained small-worldness, its Eg was significantly reduced compared to that of HCs; ii) Local topological properties show abnormal NE and DC primarily in brain regions mainly associated with the limbic system; iii) Depression scores in the OH group were negatively correlated with Eg, NE in the left amygdala, and NE in the left paracentral lobule, while NE in the MCG. R was positively correlated with executive functions/visuospatial abilities scores and clock drawing test score.

Through the analysis of the global properties of the WM network, we found that the Eg of the OH group was significantly decreased compared to the HCs group, indicating that the overall information transfer efficiency of the network was reduced in OH patients. Similar reductions in Eg have been reported in studies of depression (18), Alzheimer’s disease (24) and OH model rats (25). There was a negative correlation between Eg and depression scores in the OH group. Therefore, we speculated that this decrease in information transmission efficiency may be involved in the neuropathophysiological mechanisms of depression in OH patients. Despite these changes, the WM network in OH patients maintained small-worldness, consistent with findings in other cognitive and psychological illnesses (26, 27). This preservation indicates that, even under pathological conditions, the brain still tries to maintain a small-world network structure to support basic neural function and information processing needs.

The limbic system plays a crucial role in emotional expression and cognitive processes (28, 29). In this study, the local topological properties of the WM network showed that the NE and DC in multiple brain areas of the limbic system were decreased and the nodal local efficiency was increased in the OH group. Concurrently, OH patients exhibited lower cognitive function scores and more pronounced depressive symptoms. These findings suggest that alterations in the local topological properties of the limbic system may be integral to the neuropathophysiological mechanisms underlying cognitive and emotional abnormalities in OH. Previous studies have also found limbic system impairments in several diseases associated with cognitive impairment and depression, including Alzheimer’s disease (30), depression (31) and schizophrenia (32). This further supports the notion that limbic system impairments may affect cognitive and emotional processing in OH patients. The amygdala is a critical region within the emotional circuitry (33), playing a central role in emotion regulation (34, 35). It is closely connected with the prefrontal cortex and hippocampus, forming complex neural circuits involved in emotional regulation (36). In this study, decreased DC and NE in the left amygdala may indicate a reduced capacity for information dissemination and network stability in this region. Partial correlation analysis revealed a negative correlation between the altered topological properties of the left amygdala and depression scores, suggesting that the left amygdala may contribute significantly to emotional dysregulation in OH patients. Our findings also indicated that OH patients exhibited elevated levels of CHOL and LDL, consistent with previous research on hypothyroidism (37). This dyslipidemia is primarily attributed to the downregulation of LDL receptor expression and reduced activity of cholesterol 7α-hydroxylase due to altered thyroid hormone levels, leading to decreased hepatic clearance of LDL and impaired conversion and excretion of CHOL (38). Dyslipidemia has been associated with depression (39). In our study, both DC and NE in the left amygdala negatively correlated with LDL levels. However, in the further mediation analysis where LDL was treated as an independent variable, the NE and DC of the left amygdala were treated as mediating variables, and the HAMD-24 scores were the dependent variable, no direct or indirect mediation effects were observed. Therefore, whether lipid levels affect the emotional regulation of OH patients by influencing the NE and DC of the left amygdala requires further investigation. The MCG.R is integral in integrating cognitive functions and regulating emotional processes (40), serving as a critical region in the control of active behavior (41). We observed that NE in the MCG.R positively correlated with executive functions/visuospatial abilities, and clock drawing test score, while negatively correlated with disease duration. Therefore, it is speculated that this brain region may be a key region associated with cognitive decline in the limbic system of OH patients and affected by the duration of the disease. The temporal pole is implicated in integrating exteroceptive information and regulating interoceptive states related to social interactions (28, 42). Thyroid hormones play a crucial role in the myelination process of the central nervous system. The myelin sheath enhances the conduction velocity of nerve impulses, and insufficient FT4 levels can inhibit myelination, impairing nerve conduction (43). These findings suggest that thyroid hormone dysregulation may affect the temporal pole, leading to impaired myelination, reduced neural signal conduction velocity, and decreased NE in this region.

Furthermore, our analysis revealed decreased NE in the left angular gyrus and left precuneus among OH patients, while NE and DC were increased in the right Heschl’s gyrus. These regions are also critical nodes within the DMN, which has been implicated in facilitating a range of high-level cognitive functions (44, 45, 46). Previous studies have found that abnormalities in the DMN are widespread in depression (47), Alzheimer’s disease (48, 49), bipolar disorder (50, 51) and other disorders that cause cognitive and emotional abnormalities. This further suggests that the abnormal topological properties of WM network in multiple brain regions within the DMN may be involved in the neuropathophysiological mechanisms underlying brain impairments in OH patients.

In addition, we found that the NE of the left olfactory cortex decreased and was negatively correlated with the disease duration. The olfactory cortex is responsible for smell perception and also participates in odor discrimination and the emotional changes elicited by smells (52). Atrophy of this brain region has been observed in patients with Alzheimer’s disease (53). Therefore, we speculate that the left olfactory cortex is a vulnerable area in OH patients and is affected by disease duration. We found decreased NE in the left paracentral lobule, which negatively correlated with depression scores, suggesting that the paracentral lobule may be involved in emotional regulation processes in OH patients. Previous studies have reported volume reductions in this brain region and reduced resting-state functional connectivity in schizophrenia patients (54) and elderly depression patients (55). These findings imply that the paracentral lobule may be an important region associated with emotional abnormalities.

While this study successfully achieved non-invasive quantification of fiber connections in the brain WM network using DTI, several limitations warrant consideration. First, due to the complex architecture of WM fibers, DTI encounters difficulties in handling regions with fiber crossings and branching patterns, which may lead to incomplete fiber tracking results. Second, since this study was cross-sectional, it could not establish causal relationships. Therefore, a longitudinal study is needed to demonstrate the dynamic changes in WM in OH patients. Third, although there was a difference in gender between the two groups in this study, we included it as a covariate in the statistical analysis to minimize its impact on the research results as much as possible.

By integrating DTI with graph-theoretical analysis, this study identified significant abnormalities in the global and local topological properties of WM network in patients with OH, particularly within multiple brain regions of the limbic system and the DMN. The left amygdala and left paracentral lobule may be key regions associated with emotional regulation, while the right median and paracentral cingulate gyri may be related to cognitive dysfunction. In addition, our findings suggest that the left amygdala may be influenced by lipid levels, and the left temporal pole may be more significantly influenced by thyroid hormone levels. Collectively, these results provide novel insights into potential neuroimaging biomarkers for OH, enhancing our understanding of the disease’s neuroimaging characteristics.

## Supplementary materials



## Declaration of interest

All authors declare that there are no conflicts of interest that could be perceived as prejudicing the impartiality of the research reported.

## Funding

This work was supported by the Key Research and Development Program of Gansu Province, China (No. 23YFFA0048), the Guiding Plan Project for Scientific and Technological Development in Lanzhou City (No. 2019-ZD-97), the Gansu Traditional Chinese Medicine Management Scientific Research Project (No. GZKP-2020-36), the Foundation of Gansu Provincial Hospital (No. 24GSSYB-5), and the Outstanding Graduate Student Innovation Star Program (No. 2023CXZX-759).

## Consent statement

Consent was obtained from each patient or subject after full explanation of the purpose and nature of all procedures used.
