# Supplementary material for: Alterations of brain white matter network topological properties in overt hypothyroidism
Source: Endocr Connect. 2025 May 16;14(5):e250039. doi: 10.1530/EC-25-0039 (PMC12087277; doi:10.1530/EC-25-0039)
Supplement: Supplementary file 1 [file supplementary_materials.pdf]

Table S1. The multiple linear regression results of the OH group

		B	*β*	*t* values	*P* values	*F*	*R2*	Adjusted *R2*
NE of left amygdala								
	LDL	−0.002	−0.312	−2.279	**0.027**	5.192	0.098	0.079
DC of left amygdala								
	LDL	−0.084	−0.405	−3.070	**0.004**	9.425	0.164	0.147
NE of TPOsup.L								
	FT4	0.001	0.327	2.394	**0.021**	5.730	0.107	0.088
NE of TPOmid.L								
	FT4	0.001	0.288	2.086	**0.042**	4.353	0.083	0.064

B, non-standardized partial regression coefficient; *β*, standardized partial regression coefficient; *R2*, multiple correlation coefficient squared; the nodal efficiency of left amygdala (NE of left amygdala); the degree centrality of left amygdala (DC of left amygdala); the nodal efficiency of left temporal pole (superior temporal gyrus) (NE of TPOsup.L); the nodal efficiency of left temporal pole (middle temporal gyrus) (NE of TPOmid.L).

Table S2. The demographic data and clinical variables of the two groups (HCs and OH)

	HCs (n =86)	OH (n = 50)	χ^2/ *t* /*z* value	*P* value
Sex (M/F)	28/58	10/40	2.477 [a]	0.116
Age (years)	40.47 ± 10.57	40.08 ± 10.31	0.207[b]	0.837
Education (years)	13.67 ± 3.43	13.08 ± 3.75	0.941 [b]	0.348
TSH (mIU/L)	2.00 (1.17)	61.27 (64.52)	−9.707[c]	**< 0.001**
T3 (nmol/L)	1.54 (0.41)	1.07 (0.74)	−6.685[c]	**< 0.001**
T4 (nmol/L)	97.80 (20.61)	34.05 (40.70)	−9.642[c]	**< 0.001**
FT3 (pmol/L)	4.42 (0.67)	2.77 (1.38)	−7.680[c]	**< 0.001**
FT4 (pmol/L)	13.13 (2.06)	5.77 (2.45)	−9.397[c]	**< 0.001**
CHOL (mmol/L)	4.33 ± 0.80	5.30 ± 1.19	−5.107 [b]	**< 0.001**
TG (mmol/L）	1.11 (0.95)	1.22 (0.75)	−1.862[c]	0.063
HDL (mmol/L)	1.22 ± 0.27	1.32 ± 0.28	−2.200[b]	0.030
LDL (mmol/L)	2.54 ± 0.67	3.24 ± 0.82	−5.373[b]	**< 0.001**
TgAb (IU/ml)	1.21 (2.88)	226.17 (970.10)	−8.293[c]	**< 0.001**
TPOAb (IU/ml)	0.70 (1.32)	771.99 (820.46)	−8.567[c]	**< 0.001**

Data are presented as means ± standard deviations and median (interquartile range); a, represents the χ^2 value; b, represents the *t* value (two-sample *t*-test); c, represents the *z* value (Mann-Whitney *U* test). Abbreviations: healthy controls (HCs); overt hypothyroidism (OH); thyroid-stimulating hormone (TSH); thyroxine (T4); triiodothyronine (T3); free triiodothyronine (FT3); free thyroxine (FT4); cholesterol (CHOL); triglyceride (TG); high density lipoprotein (HDL); low density lipoprotein (LDL); anti-thyroglobulin antibodies (TgAb); thyroid peroxidase antibodies (TPOAb).

Table S3. Neuropsychological assessments of the two groups (HCs and OH)

	HCs (n =86)	OH (n = 50)	*t/z* value	*P* value
**MoCA**	26.93 ± 2.69	25.44 ± 3.29	2.877[b]	**0.005**
Subdomains of MoCA				
**Visuospatial/executive**	4.21 ± 1.05	3.90 ± 1.05	1.695[b]	0.092
Modified trail making test	1.00 (0.00)	1.00 (1.00)	−1.675[c]	0.094
Copy of the cube	1.00 (0.00)	1.00 (0.00)	−0.383[c]	0.702
Clock drawing test	2.63 ± 0.64	2.42 ± 0.67	1.829[b]	0.070
**Naming**	2.94 ± 0.23	2.90 ± 0.30	0.911[b]	0.364
**Attention**	5.71 ± 0.57	5.26 ± 1.12	2.641[b]	**0.010**
Digit span forward and backward	1.86 ± 0.33	1.83 ± 0.37	0.498[b]	0.619
Vigilance (letter tapping test)	1.00 (0.00)	1.00 (0.00)	−1.825[c]	0.068
serial sevens test	2.94 ± 0.24	2.66 ± 0.77	2.514[b]	**0.015**
**Language**	2.54 ± 0.67	2.60 ± 0.66	−4.999[b]	0.618
Sentence repetition	1.69 ± 0.57	1.75 ± 0.55	−0.576[b]	0.565
Letter fluency	1.00 (0.00)	1.00 (0.00)	−0.008[c]	0.994
**Abstraction**	1.89 ± 0.43	1.77 ± 0.42	1.562[b]	0.121
**Delayed recall**	4.00 (2.25)	3.00 (2.00)	−2.885[c]	**0.004**
**Orientation**	5.95 ± 0.21	5.96 ± 0.20	−0.213[b]	0.832
**HAMD-24**	2.00 (5.00)	6.00 (6.25)	−4.089[c]	**< 0.001**
**HAMA**	1.00 (2.00)	4.00 (7.25)	−5.142[c]	**< 0.001**

Data are presented as means ± standard deviations and median (interquartile range). b, represents the *t* value (two-sample *t*-test); c, represents the *z* value (Mann-Whitney *U* test); Abbreviations: healthy controls (HCs); overt hypothyroidism (OH); Montreal Cognitive Assessment (MoCA); Hamilton Rating Scale for Depression-24 (HAMD -24); Hamilton Anxiety Rating Scale (HAMA).

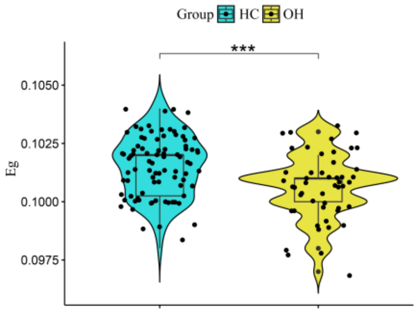

Figure S1. Intergroup differences of Eg between the two groups. * represents $P < 0.05$; ** represent $P < 0.01$; *** represent $P < 0.001$. Abbreviations: healthy controls (HCs); overt hypothyroidism (OH); global efficiency (Eg).

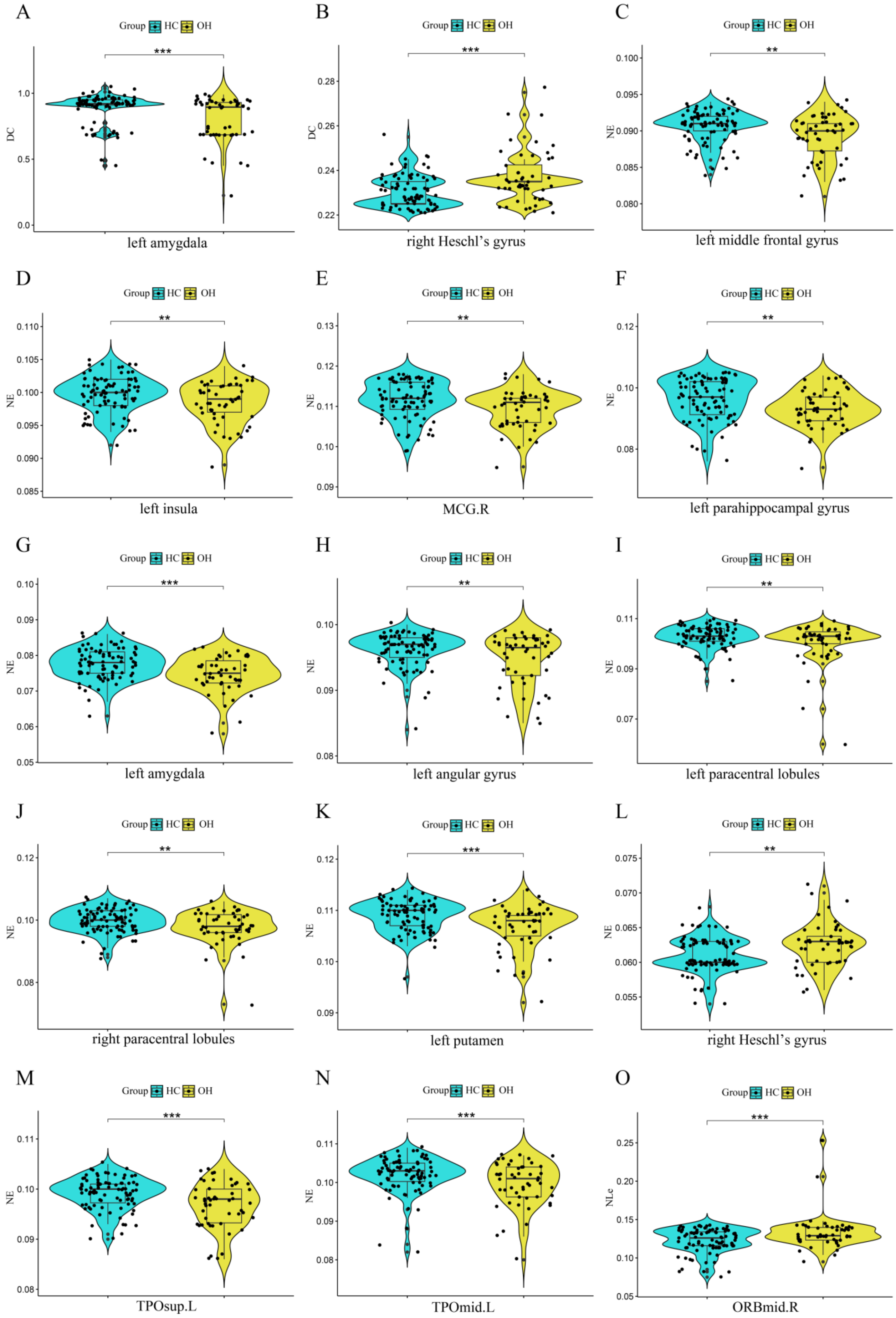
A
Group HC OH
***
DC
1.0
0.5
0.0
left amygdala
B
Group HC OH
***
DC
0.28
0.26
0.24
0.22
right Heschl's gyrus
C
Group HC OH
**
NE
0.100
0.095
0.090
0.085
0.080
left middle frontal gyrus
D
Group HC OH
**
NE
0.110
0.105
0.100
0.095
0.090
0.085
left insula
E
Group HC OH
**
NE
0.13
0.12
0.11
0.10
0.09
MCG.R
F
Group HC OH
**
NE
0.12
0.10
0.08
left parahippocampal gyrus
G
Group HC OH
***
NE
0.10
0.09
0.08
0.07
0.06
0.05
left amygdala
H
Group HC OH
**
NE
0.10
0.09
0.08
left angular gyrus
I
Group HC OH
**
NE
0.11
0.09
0.07
left paracentral lobules
J
Group HC OH
**
NE
0.12
0.10
0.08
right paracentral lobules
K
Group HC OH
***
NE
0.12
0.11
0.10
0.09
left putamen
L
Group HC OH
**
NE
0.075
0.070
0.065
0.060
0.055
right Heschl's gyrus
M
Group HC OH
***
NE
0.11
0.10
0.09
0.08
TPOsup.L
N
Group HC OH
***
NE
0.11
0.10
0.09
0.08
TPOmid.L
O
Group HC OH
***
NLe
0.25
0.20
0.15
0.10
0.05
ORBmid.R

Figure S2. (A–B) Intergroup differences of DC values of left amygdala and right Heschl's gyrus between the two groups. (C–N) Intergroup differences of NE values of MFG.L, left insula, MCG.R, left parahippocampal gyrus, left amygdala, left angular gyrus, left paracentral lobule, right paracentral lobule, left putamen, right Heschl's gyrus, TPOsup.L, and TPOmid.L between the two groups. (O) Intergroup differences of NLe value of ORBmid.R between the two groups. * represents $P < 0.05$; ** represent $P < 0.01$; *** represent $P < 0.001$. Abbreviations: healthy controls (HCs); overt hypothyroidism (OH); right median cingulate and paracingulate gyri (MCG.R); left temporal pole (superior temporal gyrus) (TPOsup.L); left temporal pole (middle temporal gyrus) (TPOmid.L); right orbital part of middle frontal gyrus (ORBmid.R); nodal efficiency (NE); degree centrality (DC); nodal local efficiency (NLe).
